# Identification of safe trocar insertion sites guided by ultrasonography to minimize epigastric vessel injuries in pediatric laparoscopic procedures

**DOI:** 10.3389/fped.2026.1769108

**Published:** 2026-05-14

**Authors:** Cemal Bilir, Zeynep Ayvat Öcal

**Affiliations:** 1 School of Medicine, Department of Pediatric Surgery, Izmir Bakircay University, Izmir, Türkiye; 2 School of Medicine, Department of Radiology, Izmir Bakircay University, Izmir, Türkiye

**Keywords:** Doppler ultrasound, inferior epigastric artery, laparoscopy, ultrasonography, vessel injuries

## Abstract

**Background & aim:**

In pediatric laparoscopy, deep epigastric vessels are among the structures most frequently injured during trocar insertion, potentially leading to significant bleeding. Although the anatomical course of these vessels has been described in adults, a systematic ultrasonographic mapping in children is lacking. The aim of this study was to determine the location of deep epigastric vessels in pediatric patients using Doppler ultrasonography and to identify safe zones for trocar placement.

**Methods:**

This study involved 90 pediatric patients who underwent color doppler ultrasound evaluation of the deep epigastric vessels before laparoscopic surgery. The deep epigastric vessels were identified at 5 equidistant levels between the xiphoid process and the symphysis pubis. Bilateral measurements of the distance of the epigastric vessels from the midline were taken at each level. Predictive analysis utilized a linear mixed model with maximum likelihood estimation.

**Results:**

The mean distances of the deep epigastric vessels to the midline at different levels in the abdominal region were 6.18 ± 0.7 cm at the right pubic symphysis, 4.64 ± 0.6 cm from the umbilicus, and 3.21 ± 0.5 cm at the level of the xiphoid. On the left, it was 6.46 ± 0.69 cm from the pubic symphysis, 4.64 ± 0.6 cm from the umbilicus, and 3.19 ± 0.5 cm at the level of the xiphoid.

**Conclusion:**

Significant variation in the distance of deep epigastric vessels from the midline in pediatric patients was observed, with no significant differences between male and female children. Consistent with the existing literature, the findings identify relatively safe avascular areas for lateral trocar placement in the pediatric population.

## Introduction

Laparoscopic surgery has become an increasingly common method for managing abdominal diseases in children (1). This approach is often preferred due to its minimally invasive nature and faster recovery time (2). However, the placement of trocars used in laparoscopic procedures poses a risk of injuring important vascular structures such as the epigastric vessels (3). Abdominal wall vessel injuries during laparoscopy have been reported to range between 0.2% and 2% (4). The vessels at risk include the superficial epigastric vessels, superficial circumflex iliac vessels, superior epigastric vessels, and inferior epigastric vessels.

The superficial epigastric and superficial circumflex iliac vessels are named for their proximity to the skin. In contrast, the superior and inferior epigastric vessels are classified as deep vessels because they are closer to the rectus fascia (5). These deep vessels are larger than superficial vessels and can lead to more severe problems if injured (5). The inferior epigastric artery originates from the external iliac or femoral artery and initially passes beneath the rectus muscle, continuing within the abdominal wall and beyond the umbilicus (6). The superior epigastric artery, on the other hand, originates from the internal thoracic artery at the level of the seventh costal cartilage and travels downward after penetrating the rectus muscle (7).

While transillumination can be useful for superficial vessels, it is ineffective for deep vessels covered by preperitoneal adipose tissue (8). Therefore, deep epigastric vessels are at risk during trocar entry. Accurate identification of the epigastric vessels and the careful placement of trocars to avoid injuring these vessels are critical factors in enhancing the safety of laparoscopic surgery. Ultrasonography enables surgeons to visualize the location of epigastric vessels and other vascular structures, allowing for the identification of the safest trocar placement sites and reduction of vessel injuries (9).

To date, there has been no study mapping the location of deep epigastric vessels in the abdominal cavity of children using Doppler ultrasonography. The aim of this study is to map the course of the deep epigastric arteries bilaterally within the abdominal wall and to identify safe zones for trocar insertion during laparoscopic surgery in the pediatric population.

## Method

This study was conducted with pediatric patients scheduled for laparoscopic surgery between July 2023 and June 2024 at İzmir Bakırçay University Çiğli Training and Research Hospital. After the study protocol was approved by the local ethics committee of İzmir Bakırçay University, patients were included in the study.

**Figure 1:**
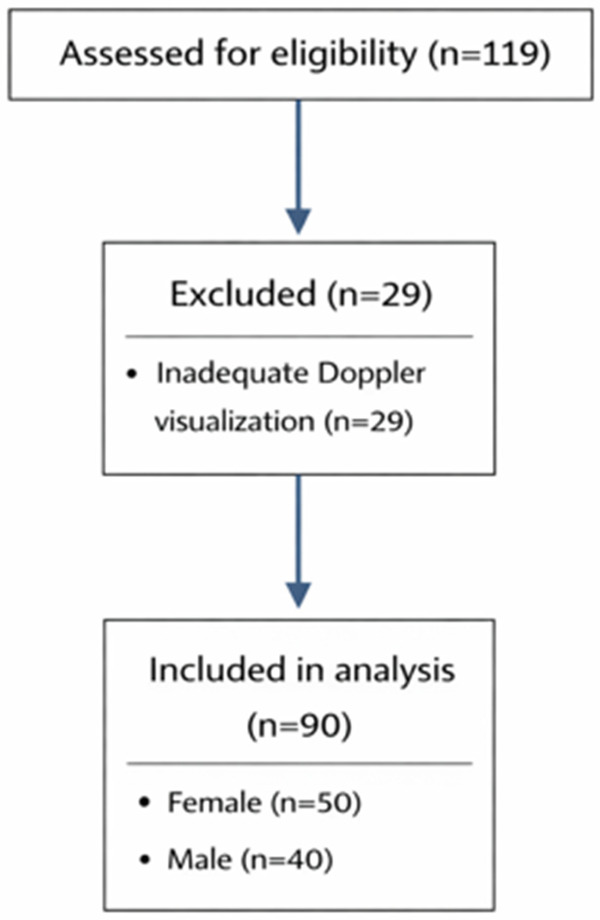
Patient selection flow diagram.

Patients with a history of transverse lower abdominal incision, as well as those with conditions such as significant skeletal deformities that could displace the epigastric vessels, large intraabdominal tumors, tense ascitic fluid, or hernias, were excluded from the study. Patients in whom the deep epigastric vessels could not be adequately visualized using Doppler ultrasonography were excluded from the analysis. The main reasons for exclusion included poor acoustic window due to bowel gas, increased subcutaneous tissue, and technical limitations during imaging. The study population consisted of patients aged 11–12 years, as laparoscopic procedures requiring trocar placement are more commonly performed in this age group in our clinical practice, allowing for more consistent ultrasonographic evaluation (Figure 1).

**Figure 2:**
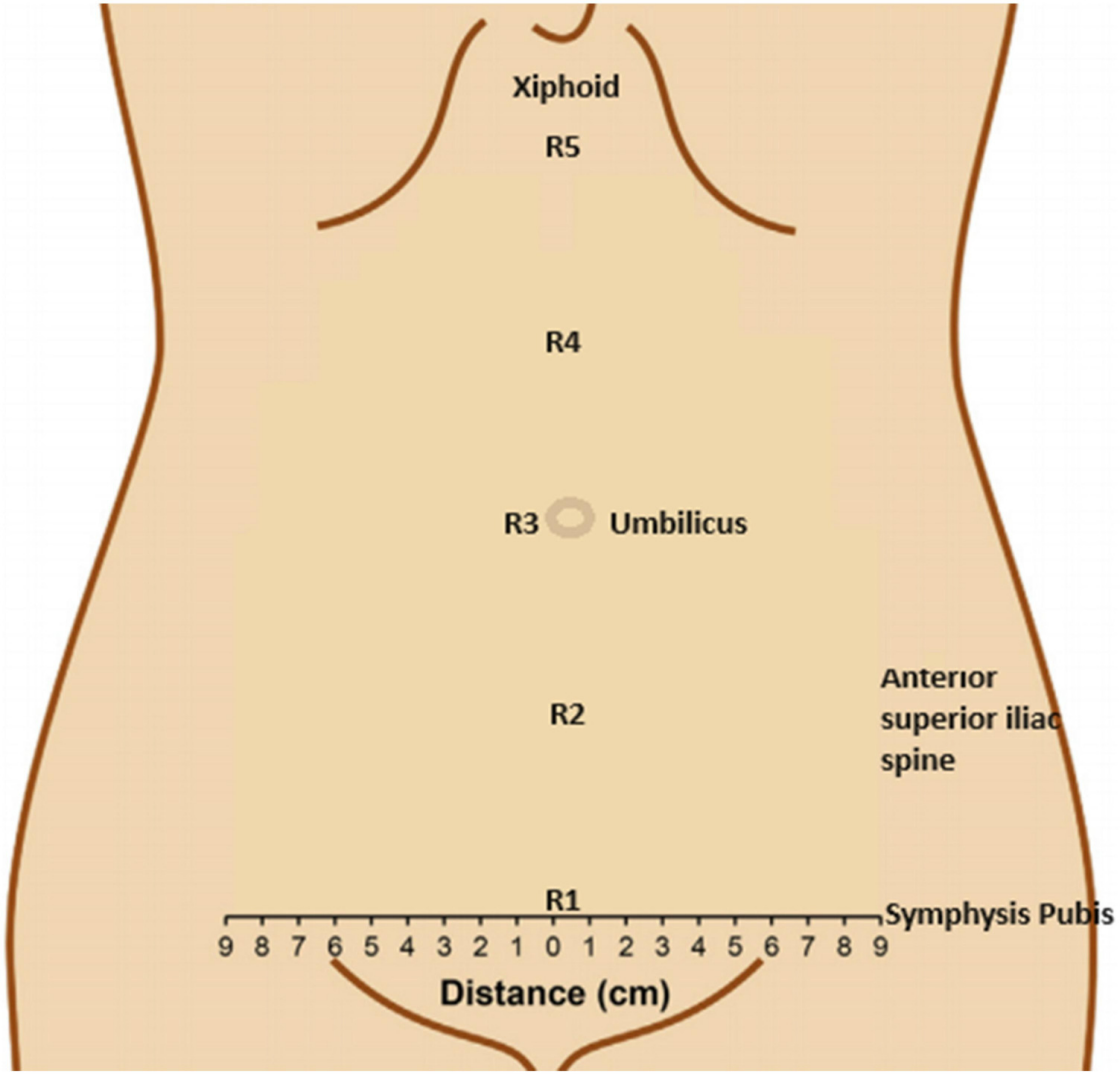
Deep epigastric vessels’ projection points on the abdominal wall.

The location of the deep epigastric vessels was mapped on the abdomen using Doppler ultrasonography at five equal levels between the xiphoid and the pubic symphysis (Figure 2). The reliability of Doppler ultrasonography for measuring the distance of the epigastric vessels has been demonstrated in previous studies. At each level, the distance of the epigastric vessels from the midline was measured bilaterally. A total of 10 measurements were taken from the right and left abdominal regions of each patient, from the xiphoid line to the pubic symphysis line.

Subsequently, the waist circumference, weight, and height of the patients were measured. Waist circumference was measured in the horizontal plane between the mid-axillary line, the lower rib edge, and the iliac crest. Height and weight were measured with participants standing barefoot and in light clothing.

To control for interoperator variability, vessel localization was performed by a single experienced vascular ultrasonography technician using a Mindray R9 ultrasound machine. Patients included in the study were marked vertically from the pubic symphysis to the xiphoid and horizontally at each level. The most lateral branch of the inferior epigastric artery and vein or superior epigastric artery and vein was marked at each level. If the superior epigastric artery was not visible at the umbilicus and xiphoid point, it was measured at the most caudal point where it could be visualized.

### Definition of safe zone

The “safe zone” for trocar placement was defined based on the distribution of the measured distances of the deep epigastric vessels from the midline. For each anatomical region, the lower boundary of the distance (mean−standard deviation) was considered to estimate the closest expected location of the vessels. Areas located medial to this boundary were considered relatively safer for trocar insertion, as they represent regions with a lower likelihood of vascular injury.

### Statistical analysis

Statistical analyses were performed using IBM SPSS Statistics version 22.0 (IBM Corp., Armonk, NY, USA). Continuous variables were expressed as mean ± standard deviation (SD). The distribution of variables was assessed using the Kolmogorov–Smirnov test.

Baseline characteristics (age, weight, and height) were compared between female and male patients using the independent-samples *t*-test.

Because each patient contributed multiple measurements (bilaterally across five anatomical regions), a linear mixed-effects model was used for the primary analysis to account for within-subject correlation. In this model, the distance of the deep epigastric vessels from the midline was defined as the dependent variable. Patient identification number was included as a random effect, while gender, side (right/left), and anatomical region (Regions 1–5) were included as fixed effects. Model parameters were estimated using maximum likelihood.

The effects of anatomical region, gender, and side were evaluated within the mixed-effects framework, and the corresponding *p*-values were derived from this model.

Post hoc pairwise comparisons between anatomical regions were performed when a significant overall effect of region was identified. These comparisons were adjusted using the Bonferroni correction. A two-sided *p*-value <0.05 was considered statistically significant.

## Results

In the study, Doppler ultrasound was used to map the deep epigastric vessels in 40 male and 50 female pediatric patients. Due to technical difficulties with imaging, 29 patients were excluded from the study. The average age of patients in the female group was 11.7 ± 1.4 years, while in the male group it was 12 ± 1.6 years (*p* = 0.54). There were no differences between the two groups in terms of height and weight.

**Figure 3:**
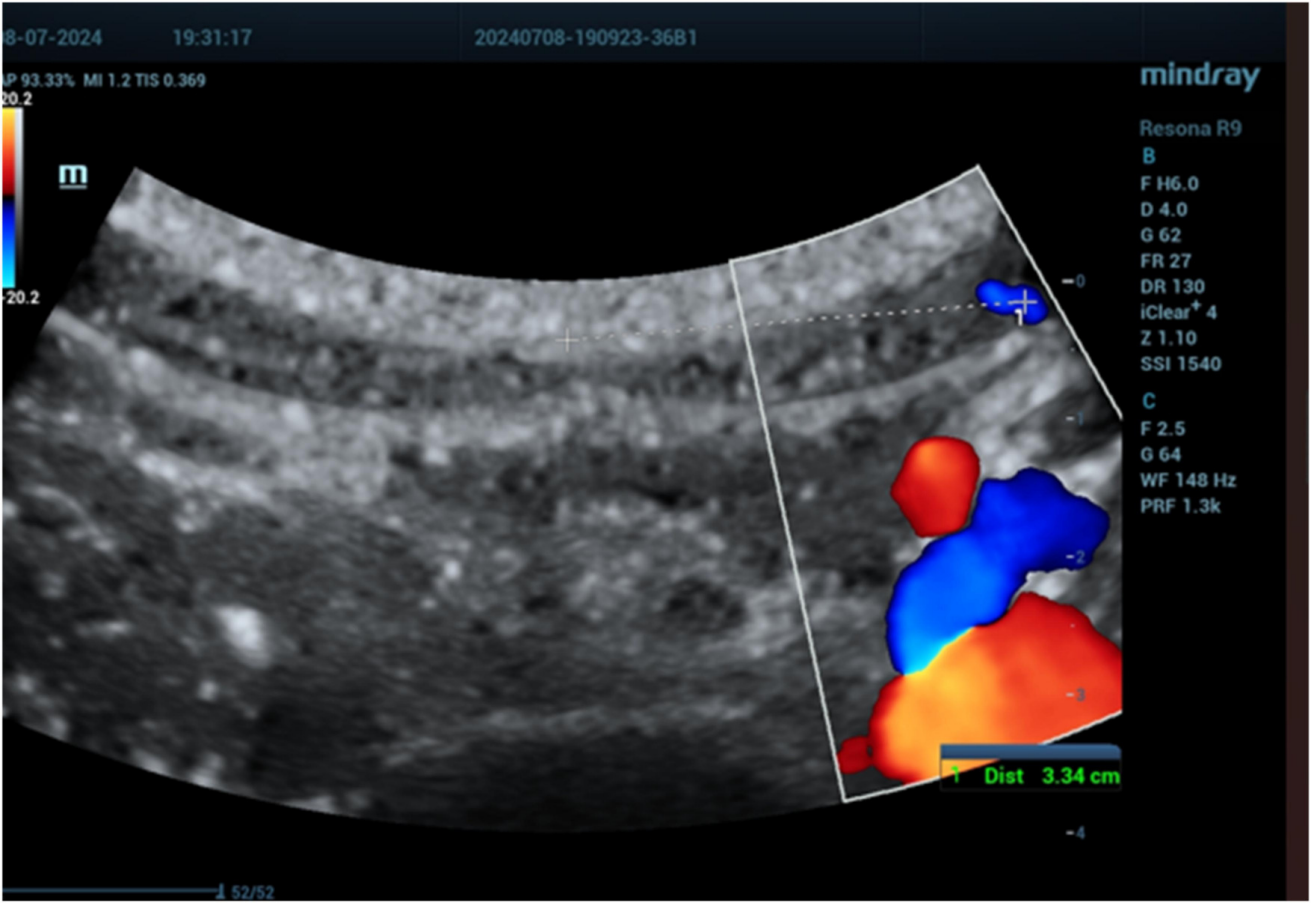
Deep epigastric vessel measurement at the symphysis pubis level (R1).

**Figure 4:**
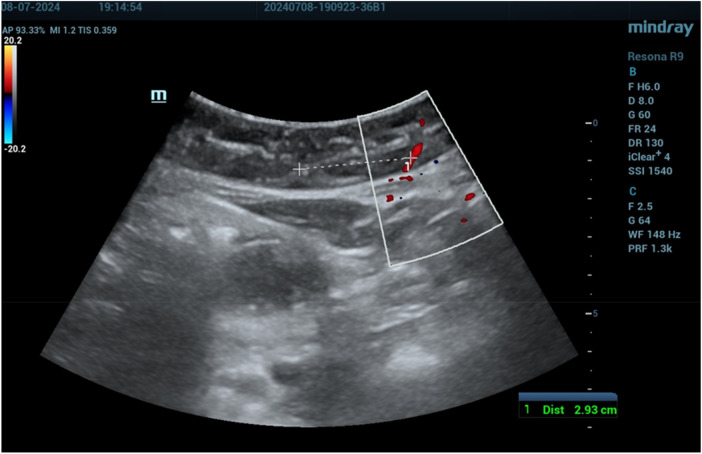
Deep epigastric vessel measurement at the umbilicus level (R3).

**Figure 5:**
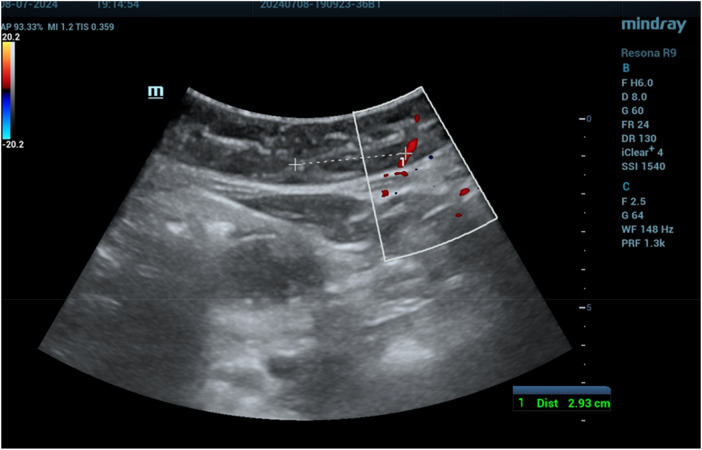
Deep epigastric vessel measurement at the xiphoid level (R5).

The mean distances of the deep epigastric vessels from the midline of the abdomen are shown in Table 1. On the right side of the abdomen, the vessels were mapped as follows: 6.18 ± 0.7 cm from the pubic symphysis zone (R1) (Figure 3), 4.64 ± 0.6 cm from the umbilicus (R3) (Figure 4), and 3.21 ± 0.5 cm at the xiphoid level (Figure 5). On the left side, the distances were 6.46 ± 0.69 cm from the pubic symphysis zone (R1), 4.68 ± 0.6 cm from the umbilicus (R3), and 3.19 ± 0.5 cm at the xiphoid level.

**Table 1:** Mean distances of the deep epigastric vessels from the midline on the abdominal wall. (mean ± sd, %95 Confidence Interval).

	Region 1	Region 2	Region 3	Region 4	Region 5
Right lateral abdominal wall, cm	6.18 ± 0.79 (6.02–6.34)	5.33 ± 0.82 (5.16–5.50)	4.64 ± 0.61 (4.51–4.77)	4.32 ± 0.77 (4.16–4.48)	3.21 ± 0.55 (3.10–3.32)

	Region 1	Region 2	Region 3	Region 4	Region 5
Left lateral abdominal wall,cm	6.48 ± 0.69 (6.34–6.62)	5.51 ± 0.74 (5.36–5.66)	4.68 ± 0.61 (4.55–4.81)	4.29 ± 0.76 (4.13–4.45)	3.19 ± 0.52 (3.08–3.30)

No significant differences were found between genders in the distance of the deep epigastric vessels from the midline at any of the measurement points (Table 2).

**Table 2:** Comparison of the projection points of deep epigastric vessels on the abdominal wall between males and females.

		Female (*n*: 50 patients)	Male (*n*: 40 patients)	*P* value
Age, years		11.7 ± 1.4	12 ± 1.6	0.54
Weight, kg		53.7 ± 9.4	52.9 ± 8.8	0.14
Height, cm		146.6 ± 11.8	149.3 ± 12.5	0.37
Region 1, cm	Right	6.1 ± 0.7	6.2 ± 0.8	0.74
	Left	6.4 ± 0.7	6.3 ± 0.6	0.65
Region 2, cm	Right	5.3 ± 0.8	5.5 ± 0.8	0.53
	Left	5.4 ± 0.7	5.6 ± 0.7	0.38
Region 3, cm	Right	4.6 ± 0.5	4.6 ± 0.6	0.21
	Left	4.4 ± 0.3	4.7 ± 0.6	0.06
Region 4, cm	Right	4.2 ± 0.8	4.3 ± 0.7	0.22
	Left	4.1 ± 08	4.4 ± 0.7	0.24
Region 5, cm	Right	3.1 ± 0.5	3.2 ± 0.5	0.28
	Left	3.3 ± 0.6	3.5 ± 0.6	0.54

Data are mean ± SD (range), in cm. Results from linear mixed-effects model.

## Discussion

Our study demonstrates that in pediatric patients, the deep epigastric vessels approach the midline of the abdomen from the level of the pubic symphysis to the xiphoid process. It also indicates that trocar insertion into the abdomen is safe if performed more than 5 cm away from the midline at the level of the umbilicus. Additionally, the projection of the epigastric vessels is similar in both females and males. As is known, injury to the abdominal wall vessels by secondary trocars has been reported in 0.3% to 2.5% of laparoscopic procedures (5). Among these, injuries to the inferior epigastric vessels account for 48% of all laparoscopic vascular injuries (10). The increase in laparoscopic surgeries has led to the development of practices aimed at reducing complications. While intraperitoneal visualization using an umbilical camera is commonly employed during laparoscopic procedures, it may not always be sufficient to reliably identify all abdominal wall vessels. In particular, superficial epigastric vessels may be visualized with transillumination, whereas the deep inferior epigastric vessels are typically not visible, as they course beneath the rectus sheath along the posterior aspect of the rectus muscle. Therefore, reliance solely on transillumination or intraperitoneal visualization may result in incomplete identification of vascular structures at risk. This limitation becomes more pronounced in patients with increased abdominal wall thickness, suboptimal pneumoperitoneum, or altered anatomy. In such cases, ultrasonographic mapping provides a valuable adjunct by enabling precise localization of both superficial and deep vessels prior to trocar insertion, thereby enhancing procedural safety.

Previous studies in the literature have focused entirely on adult populations (11). Early studies that suggested measuring from the midline to determine the location of the epigastric vessels using computed tomography images claimed that a distance of 8 cm from the midline was safe (12). Later studies using ultrasonography have reported that the inferior epigastric artery does not extend beyond 6 cm from the midline (13). Epstein and colleagues reported that the projection of the inferior epigastric artery on the abdominal wall is laterally positioned up to 9.5 cm at the umbilicus level (14). However, the study's findings were limited by the use of cadavers and their age, which constrained the generalizability of the results.

Studies on the distance of the inferior epigastric artery (IEA) from the midline show that anatomical variations can range widely. Findings from Saber and colleagues using CT scans revealed average distances of the IEA from the midline at the pubic symphysis level were 7.49 cm on the left and 7.47 cm on the right (15). In our study, the distance of the IEA from the midline at the pubic symphysis level was measured as 6.1 cm on the right and 6.4 cm on the left. These data are consistent with other findings in the literature and confirm a certain range of variation in the IEA's distance from the midline at this level.

Studies at 2 cm, 5 cm, and 7 cm above the pubic symphysis also show similar variations. Pun and colleagues found average distances of 4.9 cm on the left and 5.1 cm on the right using color Doppler ultrasound at the 5 cm level (16); these results are similar to those reported by Hurd's group from abdominal CT data (17). Additionally, Rao and colleagues found an average distance of 4 cm on the left and 4.5 cm on the right at the 7 cm level (18). In our study, at the 5 cm level above the pubic symphysis, the average distance was 5.5 cm on the left and 5.3 cm on the right. These results are consistent with other studies and indicate that the IEA's distance from the midline at this level is relatively stable.

At the umbilicus level, a wide range of variations is observed. According to Rao and colleagues, average distances at this level were 3.1 cm on the left and 3.4 cm on the right (18). Epstein and colleagues reported average distances of 5.73 cm in males and 4.79 cm in females (14). Saber and colleagues found average distances of 5.55 cm on the left and 5.88 cm on the right (15). Sriprasad and colleagues reported median distances of 4.9 cm on the left and 4.6 cm on the right (13). In our study, the distance of the IEA from the midline at the umbilicus level was found to be 4.6 cm on the right and 4.2 cm on the left. This result aligns with data from other studies, showing a small variation at this level.

The literature includes only one study focusing on the deep epigastric vessel localization at the xiphoid level, which reported that the epigastric vessels approach the xiphoid process. Similarly, in our study, we found that as the epigastric vessels progress from the umbilicus to the xiphoid, they approach the midline.

In this study, we demonstrated the location of epigastric vessels in pediatric patients. Given that our study population is a very specific age group, we are aware that our results may not be generalized. However, with the increasing frequency of laparoscopic surgical procedures in the pediatric population, the importance of vascular mapping in this group has grown to prevent vascular complications. To minimize interobserver variability and enhance measurement accuracy, we used a single experienced radiologist. Additionally, we aimed to standardize our measurements by marking the levels and midline. However, we were unable to demonstrate the perforator branches of the epigastric vessels in our measurements. One important limitation of this study is the relatively narrow age range of the study population. Although the study was designed within a pediatric framework, the included patients (11–12 years old) have anthropometric characteristics that may resemble those of small adults. Therefore, the anatomical relationships observed in this study, particularly regarding the localization of the inferior epigastric vessels, may not be directly generalizable to younger pediatric populations. Further studies including a broader age range, especially younger children, are needed to validate these findings. A relatively high number of patients were excluded due to inadequate visualization of the deep epigastric vessels. This may have introduced selection bias, as only patients with optimal imaging conditions were included in the final analysis. Therefore, the findings should be interpreted with caution, particularly when generalizing to all pediatric populations.

**Figure 6:**
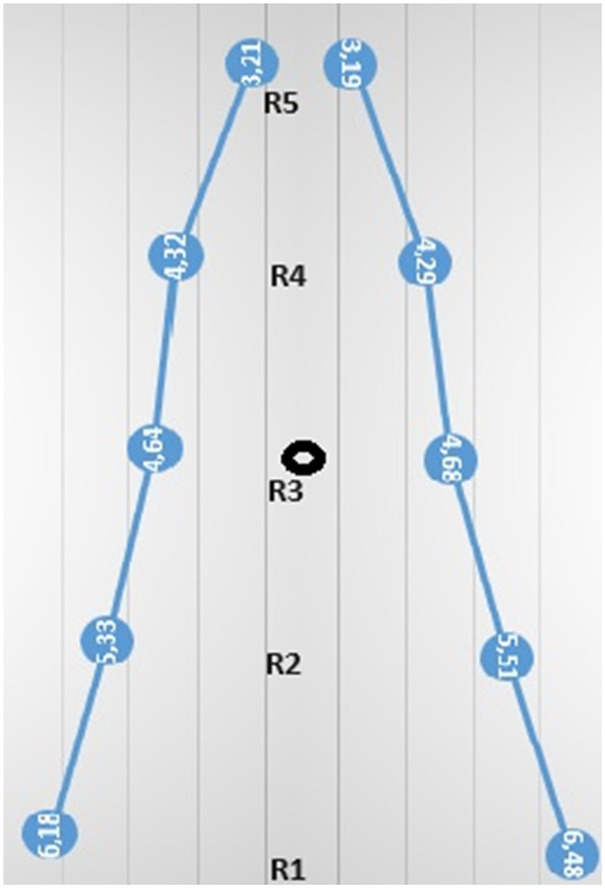
Anatomical trace of the deep epigastric vessels at different levels on the left and right abdominal walls.

In conclusion, our study shows that there is a significant range of variation in the distance of deep epigastric vessels from the midline in pediatric patients, but there are no significant differences between male and female children. Our study provides results consistent with the existing literature, showing relatively safe avascular areas for lateral trocar placement in the pediatric population (Figure 6).

## Data Availability

The original contributions presented in the study are included in the article/Supplementary Material, further inquiries can be directed to the corresponding author.
